# Prediction of Resting Energy Expenditure in Children: May Artificial Neural Networks Improve Our Accuracy?

**DOI:** 10.3390/jcm9041026

**Published:** 2020-04-05

**Authors:** Valentina De Cosmi, Alessandra Mazzocchi, Gregorio Paolo Milani, Edoardo Calderini, Silvia Scaglioni, Silvia Bettocchi, Veronica D’Oria, Thomas Langer, Giulia C. I. Spolidoro, Ludovica Leone, Alberto Battezzati, Simona Bertoli, Alessandro Leone, Ramona Silvana De Amicis, Andrea Foppiani, Carlo Agostoni, Enzo Grossi

**Affiliations:** 1 Fondazione IRCCS Ca’ Granda Ospedale Maggiore Policlinico, Pediatric Intermediate Care Unit, 20122 Milan, Italy; valentina.decosmi@gmail.com (V.D.C.); veronica.doria.vd@gmail.com (V.D.); 2 Department of Clinical Sciences and Community Health, University of Milan, 20122 Milan, Italy; alessandra.mazzocchi@unimi.it (A.M.); gregorio.milani@unimi.it (G.P.M.); giulia.spolidoro@unimi.it (G.C.I.S.); ludovica.leone@unimi.it (L.L.); 3 Pediatric Unit, Fondazione IRCCS Ca’ Granda Ospedale Maggiore Policlinico, 20122 Milan, Italy; 4 Fondazione IRCCS Ca’ Granda Ospedale Maggiore Policlinico, Anestesia e Terapia Intensiva Donna-Bambino, 20122 Milan, Italy; edoardo.calderini@policlinico.mi.it (E.C.); thomas.langer@unimi.it (T.L.); 5 Fondazione De Marchi, Department of Pediatrics, Fondazione IRCCS Ca’ Granda Ospedale Maggiore Policlinico, 20122 Milan, Italy; sscaglioni50@gmail.com; 6 Institute of Microbiology Catholic University of the Sacred Heart, 29100 Piacenza, Italy; silvia.bettocchi@unicatt.it; 7 Department of Pathophysiology and Transplantation, University of Milan, 20100 Milan, Italy; 8 International Center for the Assessment of Nutritional Status (ICANS), Department of Food Environmental and Nutritional Sciences (DeFENS), University of Milan, 20131 Milan, Italy; alberto.battezzati@unimi.it (A.B.); simona.bertoli@unimi.it (S.B.); alessandro.leone1@unimi.it (A.L.); ramona.deamicis@unimi.it (R.S.D.A.); a.foppiani@gmail.com (A.F.); 9 IRCCS Istituto Auxologico Italiano, Obesity Unit and Laboratory of Nutrition and Obesity Research, Department of Endocrine and Metabolic Diseases, 20100 Milan, Italy; 10 Villa Santa Maria Foundation, Neuropsychiatric Rehabilitation Center, Autism Unit, 22038 Tavernerio (Como), Italy; enzo.grossi@bracco.com

**Keywords:** energy expenditure, metabolism, nutrition, neural networks, children

## Abstract

The inaccuracy of resting energy expenditure (REE) prediction formulae to calculate energy metabolism in children may lead to either under- or overestimated real caloric needs with clinical consequences. The aim of this paper was to apply artificial neural networks algorithms (ANNs) to REE prediction. We enrolled 561 healthy children (2–17 years). Nutritional status was classified according to World Health Organization (WHO) criteria, and 113 were obese. REE was measured using indirect calorimetry and estimated with WHO, Harris–Benedict, Schofield, and Oxford formulae. The ANNs considered specific anthropometric data to model REE. The mean absolute error (mean ± SD) of the prediction was 95.8 ± 80.8 and was strongly correlated with REE values (*R*^2^ = 0.88). The performance of ANNs was higher in the subgroup of obese children (101 ± 91.8) with a lower grade of imprecision (5.4%). ANNs as a novel approach may give valuable information regarding energy requirements and weight management in children.

## 1. Introduction

The mainstay of malnutrition management is lifestyle modification beginning in childhood [1]. The accurate estimate of energy requirements is the first step to achieve this aim, and in children, it is mainly based on the assessment of resting energy expenditure (REE). For this purpose, indirect calorimetry (IC) is currently considered the gold standard for REE measurement, although its clinical use is limited across the world. Paucity of available calorimeters due to their costs and the related manpower, the lack of expertise in results interpretation, and of patient compliance to the exam performance are limiting factors for the application of IC in clinical practice [2]. To overcome these difficulties, several predictive equations were proposed for the estimation of REE. These formulae were investigated and applied in several contexts, showing a good reliability on a population level. However, many reports found that the disagreements between formulas and IC method on an individual level were of such a degree that their accuracy appeared unpredictable in day-to-day practice [3].

Recent data pointed out that artificial neural networks (ANN) might represent a precise and accurate method to estimate REE in obese adults [4]. ANNs are computerized algorithms resembling interactive processes of the human brain that allow one to study very complex non-linear phenomena such as biological systems [5]. The fundamental advantage of these methods is their ability to make inference at an individual level rather than at a group level [6]. The base elements of the ANN are the nodes (processing elements) and the connections. Each node has its own input, from which it receives communications from other nodes and/or from the environment, and its own output, from which it communicates with other nodes or with the environment. Moreover, each node has a function through which it transforms its own global input into an output. The connections between the nodes can modify themselves over time in a dynamic learning process, leading to the identification of new complex patterns between inputs and outputs and to the prediction of estimates about unknown data [5]. Due to these abilities, ANN has been successfully applied in medical decision support systems in many fields, such as the identification of the predictive value of risk factors to the prediction of optimal drug dosages for disease management [7,8]. However, to the best of our knowledge, no study has investigated the applicability of ANNs for REE prediction in childhood. In this study, we aimed to gauge the accuracy of ANNs for the estimation of REE in a healthy pediatric general population and to compare the accuracy of such a method with the other available estimation formulae.

## 2. Methods

### 2.1. Study Patients

We enrolled healthy children attending a primary school and the ICANS Center (Centro Internazionale per lo Studio della Composizione Corporea) based in Milan between July 2008 and March 2017. A multidisciplinary team including pediatricians, dieticians, and nutritionists completed the nutritional assessment by anthropometric measurements and performed the measurement of REE.

### 2.2. Nutritional Assessment

Body weight was measured using a gram scale, accurate to 0.1 kg, and body length with 417 SECA stadiometer (^®^ SECA Medical Measuring Systems and scales, Birmingham, UK) or a flexible but non-stretchable tape measure. Body mass index (BMI) was calculated as weight (kg)/length or height (m^2^). Z-scores for weight for age, BMI, and weight for length were calculated using the WHO Anthro and Anthro Plus^®^ software and the WHO reference charts [9]. To assess body composition, upper arm muscle area estimate (UME), upper arm fat area estimate (UFE), total upper arm area (TUA), and arm fat percentage were calculated based on anthropometric measurements [10]. Upper arm circumference (UAC) was measured using an inelastic tape measure; the midpoint between the acromial process of the scapula and the olecranon (elbow) was marked with felt-tip pen. Thus, the arm perimeter was measured with the pending member at the marked point. Biceps, triceps, subscapular, and suprailiac skinfolds were measured using a Tanner-Whitehouse caliper (Holtain Ltd., Crosswell, Crymych, Pembs, UK). Each skinfold was measured three times, and the mean value was considered for analysis.

### 2.3. Indirect Calorimetry

REE was measured in thermoneutral conditions using an open-circuit indirect calorimeter (Vmax 29^®^, Sensor Medics, Yorba Linda, CA, USA). An 8 h fasting period was recommended. Oxygen consumption (VO_2_) and carbon dioxide production (VCO_2_) were measured in spontaneous breathing. Briefly, a canopy was positioned around the child’s head, and the expired air was drawn from the hood at a fixed rate. Respiratory quotient (RQ) was calculated as VCO_2_/VO_2_ and REE using the modified Weir formula, not accounting for urinary nitrogen excretion [11]. Steady state conditions were defined as at least 5 min with less than 5% variation in RQ, less than 10% variation in VO2, and less than 10% variation in minute ventilation. Data from children who did not meet steady state or had an RQ < 0.67 or > 1.3 were excluded.

### 2.4. Prediction Formulae

Energy expenditure was estimated using the five most commonly employed formulae: the WHO formula [12], the Harris–Benedict formula [13], the Schofield formula based on weight [14], the Schofield formula based on weight and height [14], and the Oxford formula [15]. Nutritional status was defined according to WHO classification; wasting was defined as BMI of less than 2 standard deviation scores (SDS) for children ≥ 5 years of age and weight for length less than 2 SDS for children < 5 years. Obesity was defined as BMI or weight for length z-score ≥ 2 SDS for children ≥ 5 and < 5 years, respectively [16]. The study was conducted in accordance with the Declaration of Helsinki, and the protocol was approved by the Ethics Committee of Fondazione IRCCs Ca’ Granda Ospedale Maggiore Policlinico, Milan, Italy (Project identification code 135/2013), and the parents of the children gave their written informed consent.

### 2.5. Statistical Analysis

#### 2.5.1. Modelling of REE with Artificial Neural Networks (ANNs)

A physician expert in ANN analysis conducted the statistical analysis. The dataset used for ANN modeling consisted of thirteen variables: age, female gender, male gender, body weight, body height, BMI, arm circumference, biceps skinfold, triceps skinfold, TUA, UME, UFE, and arm fat percentage. Multivariate analysis was carried out with supervised ANN according to the method already adopted [17]. A subgroup analysis was also performed considering obese and underweight subjects separately.

#### 2.5.2. Auto Contractive Map System

The multi-dimensional association of strength of each variable with all other variables in a dataset was computed with the Auto Contractive Map (Auto-CM) system. This method is able to compute and graph a semantic connectivity map that (i) preserves nonlinear associations among variables, (ii) captures elusive connection schemes among clusters, and (iii) highlights complex similarities among variables. The three-layered architecture and the mathematical models of AutoCM are described elsewhere [18]. By applying the minimum spanning tree to the matrix of distances, a semantic connectivity map is generated [19,20]. The Auto-CM “spatializes” the correlation among the variables (‘closeness”), and the graph identifies only the relevant associations organizing them into a coherent picture. The “central node” is the inner node that remains after bottom-up recursively pruning away the “leaves” nodes.

#### 2.5.3. TWIST (Training with Input Selection and Testing) System

In order to cut down the number of non-relevant variables in the database (i.e., the variables that do not carry any meaningful information for the prediction task), which cause a loss in the power of our inferences, we employed a special “artificial organism” called TWIST (training with input selection and testing), which is suitably designed for sorting out the most relevant variables for the sake of prediction/classification [21,22]. The TWIST system consists of a combination of two already known systems: training/testing (T&T) and input selection (IS). The T&T system is a robust data re-sampling technique that is able to arrange the source sample into sub-samples, all of which possess a similar probability density function. In this way, the database is split into two or more sub-samples in order to train, test, and validate the ANN models as effectively as possible on the basis of the available data. The IS system is an evolutionary “wrapper” system that selects variables in order to minimize their number while preserving the actual amount of task-relevant information contained in the dataset. The combined action of these two systems allows us to increase substantially the inferential power of our ANN system while simultaneously circumventing a few major technical issues. Both systems are based on a genetic algorithm, the genetic doping algorithm (GenD) developed at Semeion Research Centre (Rome, Italy) [18].

The TWIST pre-processing singled out the variables that proved to be most significant for the prediction/classification task while at the same time producing the training set and the testing set, which were extracted from a probability distribution very close to the one that provided the best performance in the task. On the variables selected by the TWIST system, the functional approximation/prediction task was carried out by means of a supervised multi-layer perceptron with four hidden units. TWIST preprocessing produced an optimal subdivision of the records in two subsamples A and B. The subsample A included 282 records (197 males), and the subsample B included 279 records (182 males).

The protocol used for the training-test procedure was the following:

1. In the first run, subset A is used as the training set and subset B as the testing set.

2. Application of ANN on the training set. In this phase, the ANN learns to associate the input variables with those indicated as targets.

3. At the end of the training phase, the weights matrix produced by the algorithm is saved and frozen together with all of the other parameters used for the training.

4. The testing set is then shown to a virgin twin (same architecture and base parameters) ANN with the same weights’ matrix of the trained ANN, acting as the final classifier. This operation takes place for all records in testing each, and results (right or wrong classification) are not communicated to the classifier. This allows one to assess the generalization ability of the trained ANN.

5. In a second run, another virgin ANN is applied to subset B, which is used as a training subset, and then to subset A, which is used as a testing subset.

6. Therefore, the results in in below figures and tables are relevant to two sequences of training testing protocol: A–B and B–A.

The accuracy results were expressed as the average of results obtained in the two independent testing sets. The REE value predicted by ANN was compared with the REE measured with IC by univariate linear regression.

The mean absolute error (MAE), i.e., the mean of the absolute difference between the predicted and the actual values, the root mean square error (RMSE), the real error, the normalized mean square error, the Kendall’s Tau Index, the Pearson coefficient of determination (*r*^2^), the paired Student T-test, the squared correlation index, and the linear correlation index were used to measure the predictive accuracy of ANN when appropriate. Data are given as mean and standard deviation, absolute or percentile values. Significance was assumed when *p* < 0.001, taking into account the existence of multiple tests. Analyses were performed using SPSS 20.0 (Statistical Package for Social Science. Inc., Chicago, IL, USA). The same fitting was carried out with the five equations in the study.

## 3. Results

### 3.1. Characteristics of the Study Population

**Figure 1:**
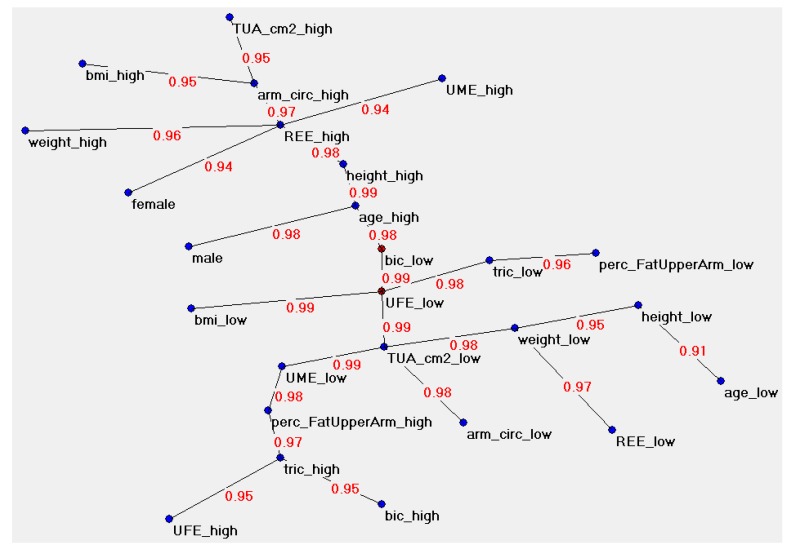
Semantic connectivity map of the 13 variables used for artificial neural network (ANN) modeling. Semantic connectivity map of the variables under study in the study group with the Auto Contractive Map (Auto-CM) system. The values on the arcs of the graph indicate the strength of the connection, measured on a scale ranging from zero to 1. TUA: total upper arm area; UME: upper arm muscle area estimate; UFE: upper arm fat area estimate; TUA: total upper arm area; perc_fat_upperarm: arm fat percentage; arm_circ: arm circumference; tric: triceps skinfold; bic: biceps skinfold; REE: resting energy expenditure.

A total of 561 consecutive subjects (379 boys, 67.5%) aged 2 to 17 years were studied. The anthropometric and the metabolic measurements of the patients are given in Table 1. Figure 1 is visual mapping of the complex web of connection schemes among variables and the principal hubs of the system, simplifying the detection of the variables that play a key role in the graph. It shows, among the full spectrum of possible ways to connect the variables in a tree, the shortest combination. The distances among variables reflect their bonding strength (weights) [18,23].

### 3.2. Fitting of REE with Artificial Neural Networks

**Figure 2:**
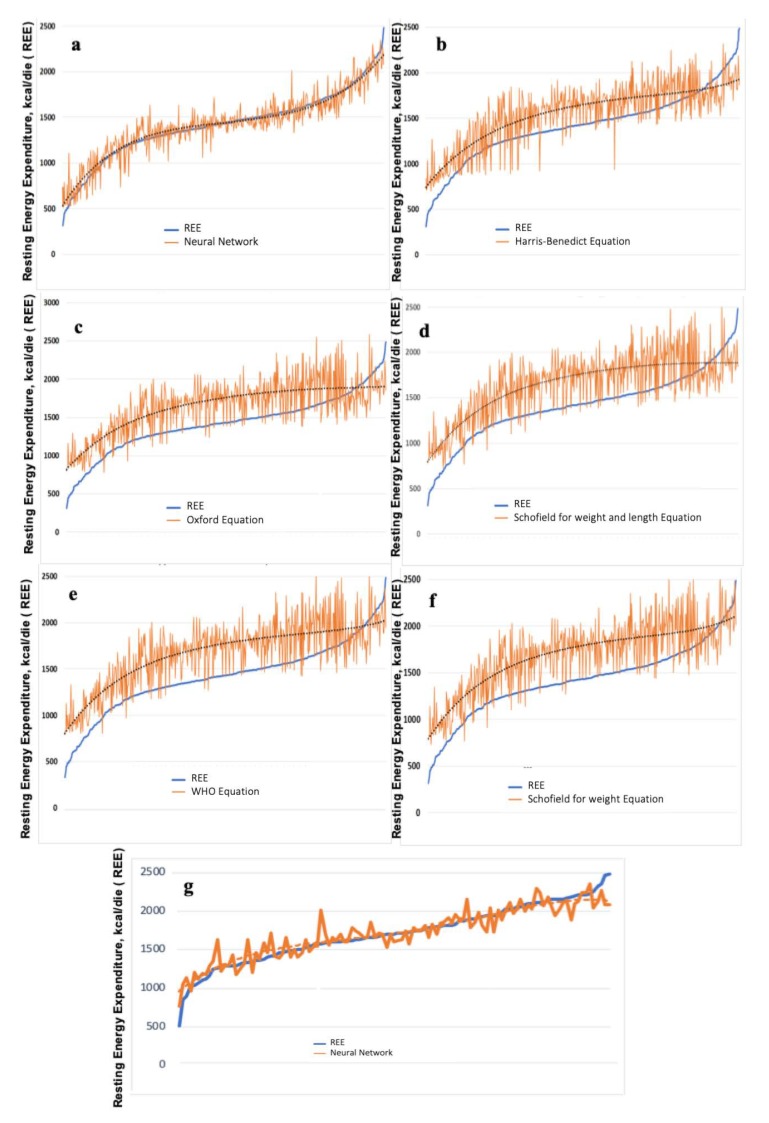
True REE approximation in total population and in obese subgroup. Approximation with neural networks (**a**) and the comparative results obtained with Harris–Benedict (**b**), Oxford (**c**), Schofield for weight and length (**d**), WHO (**e**), and Schofield for weight equations (**f**). Prediction of REE in obese children with the best ANN (**g**). The blue line expresses the true REE values, the orange line is the corresponding fitting of the method under evaluation, and the dotted line is the tendency line described by a five-degree polynomial equation.

The TWIST system selected seven variables carrying the maximal amount of information to build up a predictive model and precisely: age, female gender, weight, BMI, TUA, UME, and arm fat percentage. The final model, based on these seven variables, expressed a functional approximation of the actual REE value within a protocol based on a bipartite division of the dataset: training set sub-sample (*n* = 282) and testing sub-sample (*n* = 279). The five equations appeared to systematically overestimate the true REE value, but in the last part, true REE reached higher values in which the opposite happened with underestimation. It should be noted how the neural network tendency line appeared to be almost superimposed to the true REE values curve (Figure 2).

### 3.3. Comparative Statistics between Tests in the Study

The modeling obtained by an ANN with the same architecture trained on the same dataset including only age, weight, height, and sex reached the following predictive performance: average absolute error = 101.75 (SD 90.89) calories. The modeling obtained by the average of two independent ANNs reached an average absolute error of 95.88 calories with a *R*^2^ = 0.88 (Table 2). The *p*-value of the paired *T*-test = 0.13, suggesting that the main improvement in accuracy was related to non-linear modeling rather than the addition of other constants. The comparative values obtained with the five equations were markedly worse. The best equation in term of absolute error resulted from the Harris–Benedict, with an average absolute error of 224.16 calories, while the best equation in terms of linear correlation resulted from the Schofield for weight with a *R*^2^ value of 0.624.

The output with ANN was not statistically different from true REE (*p* = 0.295), while all equation output sets were different from a statistical point of view with an extremely high p-value (*p* < 0.0001) (Table 2). Multiple linear correlation matrix of the tests under study is shown in Table 3. The table shows how the five equation values were strongly correlated between them and poorly correlated with neural networks values and true REE values. The latter two values set at variance were strongly correlated.

### 3.4. Obese Subjects

A total of 113 subjects (48 males, 42.5%) were obese with an average BMI z-score of 2.28 (range = 2.0–3.59). In this subsample, the performance of different models was generally better in comparison with the general population. The absolute values for measured REE were, on average, 1708.6 (SD 369.2). Neural networks gave excellent results with a mean absolute error significantly lower than other models such as in the general population (Table 2). Figure 2 (panel g) shows the prediction of REE obtained with the best ANNs in this subgroup. The goodness of fit was homogeneous along the entire arch of values, with the exception of extreme high values. All the models showed lower imprecision in obese subjects and higher imprecision in underweight subjects. Neural networks modeling allowed a strong reduction in imprecision compared with standard equations in all three subgroups.

## 4. Discussion

The results of this study provide new insights derived from the ANN approach to the REE estimation in children. The prediction of REE by ANNs gave the lower mean absolute error (that is, the lowest degree of imprecision) in comparison with the five equations here considered, and it was strongly correlated with REE values as directly measured. When considering the subgroup of obese children, the performance of ANN was even better, and the grade of imprecision was lower (5.4%). Available prediction formulae were derived from populations with different nutrition habits, lifestyle (less sedentary), and body composition (higher percentage of fat free mass), in accordance with secular trends, especially the Harris–Benedict that was formulated in the early 1920s [24]. Consequently, methods and conclusions of these formulae today appear valid but might be not error free. Accordingly, there is still a lack of consensus in defining the most appropriate equation for calculating REE in children. Many studies have been conducted in hospital settings [25]. During chronic and acute illness, REE may be influenced by factors related to the clinical condition. Any state of disease may directly or indirectly alter components of energy expenditure with marked effects on nutritional status [3]. In a previous work, we evaluated the accuracy of the same five predictive formulae in 236 ill children. We found that formulas were not yet accurate at the population level but were enough at the individual level, with the consequential risk of indicating underfeeding or overfeeding [3]. Comparably, a cross-sectional study with the aim to assess the performance of 23 REE equations in patients with cancer, categorized by BMI class, cancer types, and cancer stage, found that all equations have wide limits of agreement, i.e., poor individual agreement, and bias frequently correlated to age and fat mass (FM) [26]. Another study testing the validity of equations in obese children and adolescents showed that all differ from measured REE, have a large number of errors, and over- or underestimate the real REE [27]. Similarly, Maffeis et al. and Molnar et al. indicated that REE prediction equations overestimated REE by up to 20% in their cohorts of 130 obese and nonobese prepubertal children aged 6 to 10 years and 371 children aged 10 to 16 years, respectively [28,29].

The results of the present study are relevant, since childhood obesity represents a major public health issue around the world with huge impact on actual and future health of young people. In addition, these findings, if confirmed, also support the hypothesis that current dietary recommendations mostly based on prediction formulae might constitute indications towards overfeeding, as suggested by the use of the double-labeled water method in young children [30]. On the other hand, the prediction of metabolic needs in pediatric settings is important to support child growth as well as avoiding nutritional imbalances. Indirect calorimetry is highly technical and expensive, two factors that may limit its use. Recently, some handheld devices were developed as alternative tools. Even if some studies have found that these instruments are reliable and valid for the measurement of REE in adults and children, their validity has already been questioned [31,32,33,34]. Machine learning algorithms are based on mathematical-computational methods to learn information directly from data without mathematical models and predetermined equations. We here highlight the novelty related to ANN methods, since these algorithms consider for the calculation of REE more specific anthropometric data such as TUA, UME, and arm fat percentage rather than only weight and height as common formulae. An in-depth evaluation focusing on the associations between body composition and REE might yield valuable information regarding energy requirements and weight management in children. The possible application of an easy-to-use equation in the day-by-day practice of weight management interventions for children and adolescents is quite attractive indeed.

This paper is the first one to challenge the REE prediction in healthy children with the ANN approach. REE was measured in a large set of children of different ages (2–17 years). Some concerns may be raised considering the IC advices may rely on the same prediction equations to yield the final calculations of REE. Moreover, since the measurement of skinfold thickness to assess the body composition depends on the experience and the skill of the examiners, further resolution may be provided by more sophisticated measures of body composition.

## 5. Conclusions

ANNs may provide accurate estimates of REE in healthy children, this representing new and valid alternatives to simple IC in the day-by-day clinical practice. Future studies should investigate the potential of this approach to develop personalized dietary interventions for preventive and/or therapeutic purposes [35].

## Figures and Tables

**Table 1:** Anthropometric and metabolic measurements of the study population.

	Total Population	
	Mean	SD
Age, years	13.0	3.5
Weight, kg	62.8	23.0
Height, cm	156.5	18.6
BMI	24.6	5.9
Body mass index z-score	1.1	1.1
Arm circumference, cm	28.7	5.7
Biceps skinfold, mm	13.3	6.6
Triceps skinfold, mm	22.5	9.0
Subscapular skinfold, mm	21.9	11.5
Suprailiac skinfold, mm	29.0	13.7
z-score weight for height	0.8	1.4
z-score weight for age	0.4	1.2
z-score height for age	0.4	1.2
Fat mass, kg	18.8	9.1
Free fat mass, kg	43.7	16.0
Total upper arm area, cm^2^	68.1	25.8
Upper arm muscle area estimate, cm^2^	33.8	12.1
Upper arm fat area estimate, cm^2^	34.4	18.4
Fat upper arm, %	47.8	13.5
VO_2_, L/min	0.20	0.05
VCO_2_, L/min	0.17	0.04
RQ	0.83	0.07
Resting energy expenditure, kcal/die	1417.6	368.5
Harris–Benedict energy expenditure, kcal/die	1554.2	337.2
WHO energy expenditure, kcal/die	1673.6	354.1
Schofield for weight and length energy expenditure, kcal/die	1649.4	348.1
Schofield for weight energy expenditure, kcal/die	1689.5	371.1
Oxford energy expenditure, kcal/die	1649.9	351.0

**Table 2:** Fitting performances of true REE by methods under study and statistical comparison of fitting methods with paired Student *T* test.

Overall Group (*N* = 561), Mean REE = 1147							
Fitting Method	Absolute Energy Expenditure	Absolute Error		Imprecision %	Pearson *R*^2^	*T* Statistics	*P*-Value (Two Tails)
	Mean	Mean	SD				
Neural networks	1423.14	95.88	80.86	6.80	0.88	−1.04	0.295
**Equations**							
Harris–Benedict	1554.20	224.16	137.13	15.80	0.03	−7.13	<0.0001
WHO	1673.55	300.81	180.80	21.2	0.59	25.23	<0.0001
Schofield weight and length	1649.44	300.69	178.61	21.2	0.53	20.96	<0.0001
Schofield weight	1689.51	306.93	191.30	21.7	0.62	26.99	<0.0001
Oxford	1649.93	305.56	176.29	21.6	0.52	20.81	<0.0001
**Underweight (*N* = 16), Mean REE = 1006.4**							
Neural networks		109.8	63.6	10.9			
**Equations**							
Harris–Benedict		231.2	131.4	23.1			
WHO		262.1	131.1	26.0			
Schofield weight and length		263.5	153.7	26.2			
Schofield weight		262.9	136.7	26.1			
Oxford		252.0	117.0	25.0			
**Obese (*N* = 113), Mean REE = 1708.6**							
Neural networks		101.0	91.8	5.4			
**Equations**							
Harris–Benedict		220.7	150.0	8.8			
WHO		296.6	217.6	12.7			
Schofield weight and length		287.6	205.3	12.0			
Schofield weight		288.0	233.0	13.6			
Oxford		311.1	215.9	12.6			

**Table 3:** Matrix of linear correlation among equations’ outputs each other.

	Harris–Benedict Energy Expenditure	WHO Energy Expenditure	Schofield for Weight and Length Energy Expenditure	Schofield for Weight Energy Expenditure	Oxford Energy Expenditure	Best Neural Network	Resting Energy Expenditure
Harris–Benedict energy expenditure	1						
WHO energy expenditure	0.815	1					
Schofield for weight and length energy expenditure	0.864	0.953	1				
Schofield for weight energy expenditure	0.786	0.980	0.949	1			
Oxford energy expenditure	0.835	0.968	0.975	0.954	1		
Best neural network	0.034	−0.052	0.009	−0.046	0.016	1	
Resting energy expenditure	−0.076	−0.176	−0.099	−0.173	−0.094	0.940	1
